# Hull and Aerial Holonomic Propulsion System Design for Optimal Underwater Sensor Positioning in Autonomous Surface Vessels

**DOI:** 10.3390/s21020571

**Published:** 2021-01-15

**Authors:** Bruno A. Regina, Leonardo M. Honório, Antônio A. N. Pancoti, Mathaus F. Silva, Murillo F. Santos, Vitor M. L. Lopes, Accacio F. Santos Neto, Luis G. F. Westin

**Affiliations:** 1 Faculty of Engineering, Federal University of Juiz de Fora, Juiz de Fora 36036-110, Brazil; almeida.bruno@engenharia.ufjf.br (B.A.R.); antonio.pancoti@engenharia.ufjf.br (A.A.N.P.); mathaus.silva@engenharia.ufjf.br (M.F.S.); vitor.mainenti@ufjf.edu.br (V.M.L.L.); 2 Department of Electroelectronics, CEFET-MG, Leopoldina 30.510-000, Brazil; murillo@leopoldina.cefetmg.br (M.F.S.); accacio@cefetmg.br (A.F.S.N.); 3 Itapebi Geração de Energia S.A, Neoenergia, Rio de Janeiro 22210-030, Brazil; lwestin@neoenergia.com

**Keywords:** 3DOF ASV, optimal sensor allocation, optimal hull design, shallow water operation

## Abstract

Acoustic Doppler Current Profiler (ADCP) sensors measure water inflows and are essential to evaluate the Flow Curve (FC) of rivers. The FC is used to calibrate hydrological models responsible for planning the electrical dispatch of all power plants in several countries. Therefore, errors in those measures propagate to the final energy cost evaluation. One problem regarding this sensor is its positioning on the vessel. If placed on the bow, it becomes exposed to flowing obstacles, and if it is installed on the stern, the redirected water from the boat and its propulsion system change the sensor readings. To improve the sensor readings, this paper proposes the design of a catamaran-like Autonomous Surface Vessel (ASV) with an optimized hull design, aerial propulsion, and optimal sensor placement to keep them protected and precise, allowing inspections in critical areas such as ultra-shallow waters and mangroves.

## 1. Introduction

In countries where hydroelectric power is the basis of their energetic matrix, water management is a critical issue in dispatch planning. Mathematical models are calibrated based on rain forecast and the impact of the incoming water on the reservoirs, providing tools to define the dispatch of all power plants in the grid, considering both economic and safety constraints. The result of the given planning has a significant impact on energy prices, the environment, and downstream security. There are several critical factors in this decision-making process; however, one major issue is to determine water availability by using mathematical models. As a consequence, another key issue is to determine the precise amount and quality of the water fueling the hydroelectric reservoirs [1,2] to optimize the entire plant control and energy production [3]. This measure is provided by the flow curve metric, and although it presents a significant impact on the energy prices, it is usually evaluated by acoustic Doppler current profile sensors embedded in human-operated boats without any specific procedure to optimize these readings [4].

Considering the evolution of water measurement missions, the literature shows that, in the last few decades, the demands for the development of activities by aquatic robots have increased considerably, as the need for people on the boats can make the work difficult in some environments [5]. Autonomous Surface Vessels (ASVs) can, in fact, avoid these circumstances and automatically perform observation tasks with the aid of their sensors. By definition, they are robotic vehicles for water surface operation, whether in rivers, lakes, seas, or oceans, for data collection, recording, and research, for example [6].

Considering that they can be smaller and have reduced weight when compared to manned vessels, they are often particularly useful for collecting data without disturbing the environment such as in shallow water applications, swamps, estuaries, mangroves, lagoons, and coral reefs [5,7].

In these environments, the vessel’s submerged area and propulsion system’s disturbance must be minimized to improve safety and sensor measurements. Furthermore, these characteristics may also improve the vehicle’s maneuverability, reducing contact with underwater or floating objects, increasing the accessibility to critical areas [8]. As a result of these needs, studies and research in the allocation and integration of sensors and components for ASVs become increasingly important for shallow water navigation, as the choice of equipment and their respective positions directly interfere in the vessel submerged area and in the waveform.

Raimondi et al. [9] proposed a nautical remote-controlled vehicle for surface navigation with innovative features, called Semi-Immersible (SI-ASV). It is used for applications in ports, lake monitoring, organic marine fish, hydrography, geology/geophysics, oceanography, underwater acoustics, and environmental monitoring with particular attention to climate change impact indicators. The electric power vehicle is coupled with jet propulsion, which makes it possible to navigate in shallow waters or sandbars. The vehicle was shown to be reliable and applicable to the environment presented, but this study lacked a comparison between the designed and practical results.

Giordano et al. [10] developed an ASV prototype for bathymetry surveys, named MicroVeGA, which is able to navigate in shallow waters, where traditional boats cannot access. Results were presented considering two prototypes, basically changing the shape of the ASV’s hulls, and experimental tests were performed in critical areas, including submerged archaeological remains that produce rapid changes of the depth values. The experiments confirmed that the sensor integration improved the instrument performance and survey accuracy with reasonable analysis between the design and practical results. In these cases, the propulsion motors were submerged, placed under the hull.

Li et al. [11] presented the design of the WC-ASV for automatic measurement of water-leaving reflectance in shallow waters. The integration of various sensors allowed some functions, such as remote control, status monitoring, and automatic obstacle avoidance. It is important to remark that it weighted 350 kg in fully loaded conditions and its propellers were located under the hull. The results showed that the WC-ASV can effectively ensure the consistency of data deviation, replacing the manual observation and ensuring the safety of in situ observation staff. Furthermore, the WC-ASV could fully automate the collection and measurement of the meteorological parameters, water spectrum, quality, and samples.

Despite all the work dedicated to project-specific designs for ASVs, there are still several unattended requirements such as navigation in ultra-shallow waters and redirecting the water flow generated by the vessel’s hull to mitigate interference in the measurements. Then, in this context, this work proposes the allocation and integration of sensors and equipment in an over-actuated ASV called AERO4RIVER. This vessel is designed specifically for the navigation and monitoring of ultra-shallow waters, with a minimum water depth of 0.15 m, mainly due to the aerial and azimuth propeller positioning. The approach considers three steps: first, an initial hull design analysis to ensure that all the mission requirements have been met; a second one regards an in-depth Computational Fluid Dynamics (CFD) design to study the hull behavior considering several situations (this step will provide the optimum position and constructive parameters for dynamic control); finally, practical tests compare the similarity between the design and the real boat, also describing the significant decrease of the submerged areas and their interference in the displacements and measurements. Therefore, the main contributions of this work are: a new 3DOF ASV topology design, an areal azimuth propulsion system for shallow waters, an optimal sensor placement analysis, and an approach to provide a good a *priori* parameter estimation. These improvements will help autonomous mission control [12,13], data acquisition, and safety. Moreover, considering the most recent advances in the ASV field [14,15,16,17,18,19,20], the proposed topology is new.

This work is organized as follows: Section 2 describes the ASV AERO4RIVER, presenting its main constructive characteristics, the innovative air propulsion system, and the vessel mathematical modeling; Section 3 gives a study to define the shape of the most suitable hulls for the ASV design, where different shapes are evaluated qualitatively in a CFD analysis; Section 4 presents the CFD studies carried out to define the ADCP positioning, as well as comparisons with the practical results; finally, Section 6 concludes the work.

## 2. ASV Project Requirements

The developed vehicle, named AERO4RIVER, has different constructive characteristics when compared to conventional catamarans. For practical purposes, its design is focused on the following requirements: it must be a compact vessel to facilitate transportation; it should support different types of embedded sensors, including the ADCP; it must be able to navigate in a water column as low as 15 cm; it must overcome obstacles such as algae, twigs, and small logs; it should enable three Degree of Freedom (DoF) navigation, featuring fully controllable movements in the surge, sway, and yaw; it must support a total buoyancy of at least 20 kg, the initial estimate for structural and sensor weight; it should be able to reach speeds of up to 3 m/s.

Its propulsion configuration uses four aerial thrusters positioned at the top of the hull, with their rotation axes parallel to the water surface. This configuration is also efficient when used alongside submerged sensors, as the motors do not influence the underwater measurements, contributing to noise in the aquatic environment or excessive water displacement, for example. Another advantage is that the propeller is safe from algae being stuck in it or even from suffering damage by hitting rocks, branches, or other debris usually found in shallow water environments.

The simple hull geometry serves multiple purposes. It can be more easily manufactured and modeled in CFD software due to the lack of a moving rudder or sharp stern angles. The ASV can also move more freely in waters where there is floating debris or river bottom rocks, as the smooth surface prevents objects from clinging to it.

### 2.1. Kinematic and Dynamic Model

Kinematic and dynamic modeling is a very important step to design precise controllers [21,22]. Commonly, the ASVs has six DoFs [23], where horizontal motions in the vehicle longitudinal direction are called the surge. Regarding horizontal motions in the vehicle’s orthogonal direction, they are called the sway. Movements around its vertical axis are called the yaw. The other three DoFs are the roll and pitch angles and the vertical motion (heave). For simplicity, they will be disregarded on the assumption of their minor influences on the vehicle’s maneuvering dynamics.

According to the literature, the marine vehicle nomenclature is traditionally expressed as: $\eta ={\left[x,y,\psi \right]}^{T}$ representing inertial (x,y) and angular (ψ) positions in the vehicle inertial frame $F^{I}$; $\nu ={\left[u,\upsilon ,r\right]}^{T}$ being the linear (u,υ) and angular (r) velocities of the body-fixed frame $F^{BF}$ [24].

The general ASV dynamics and kinematics modeling without disturbances is represented as follows [24]:

(1) $$\begin{array}{ccc} M\dot{\nu }+C_{RB}\left(\nu \right)\nu +N\left(\nu_{r}\right)\nu_{r} & = & \tau \end{array}$$

(2) $$\begin{array}{ccc} \dot{\eta } & = & J(\eta )\nu \end{array}$$

where $M\in R^{3}$ represents the inertia matrix, $C_{RB}\left(\nu \right)\in R^{3}$ is the rigid-body Coriolis and centripetal matrix, $N(\nu_{r})\in R^{3}$ consists of the terms of added mass of Coriolis and centripetal force together with the terms of hydrodynamic damping ($\nu_{r}$ consists of the relative speed between the movement of the vehicle and the current (rivers, oceans, etc.)), $\tau \in R^{3}$ is the vector of forces and moments generated by the thrusters, $J(\eta )\in R^{3}$ is the Jacobian matrix, the first portion of $N(\nu_{r})$ represents the added mass Coriolis effect, the second one being the linear characteristic damping phenomenon, and finally, the third one is the non-linear damping result in the quadratic drag approximation. Furthermore, the 3DoF model can be found in Fossen [23].

**Figure 1:**
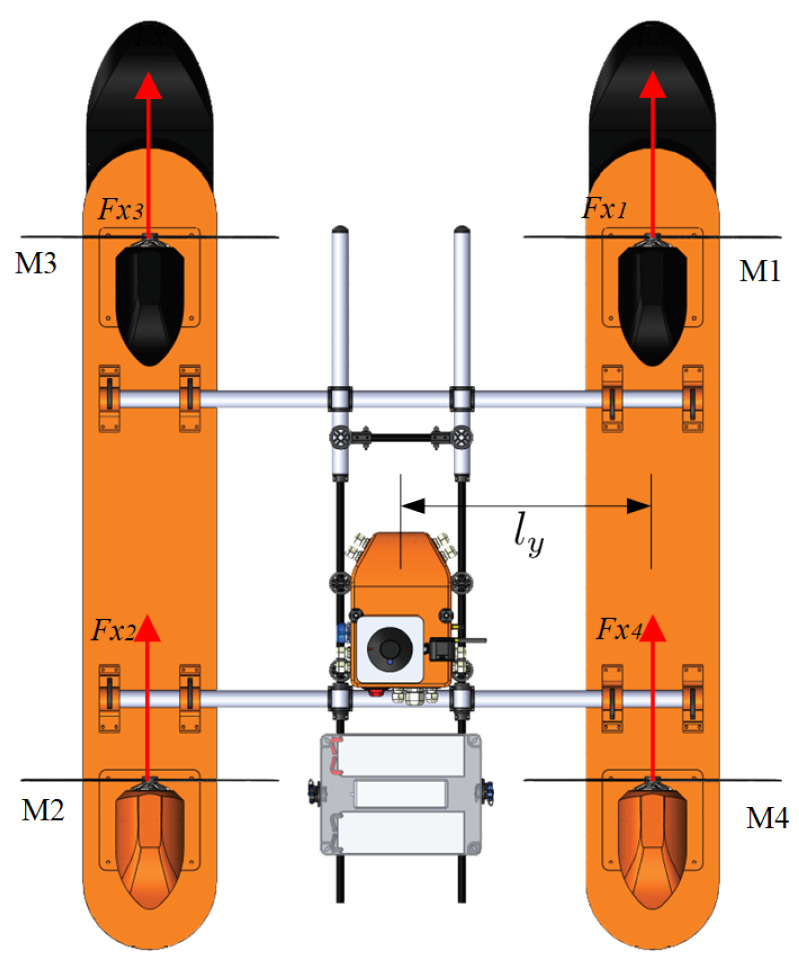
Catamaran-like boat with azimuth propulsion system.

The general design is shown in Figure 1, where the boat is represented by a catamaran-like shape with four individual azimuth propulsion systems.

**Figure 2:**
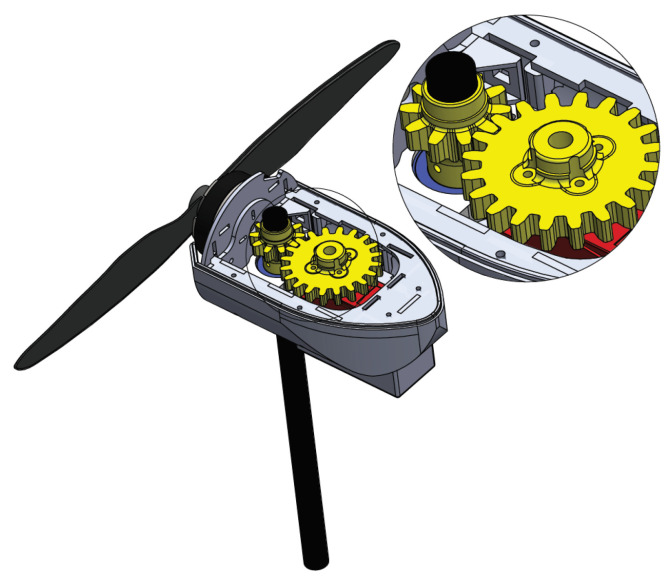
Motor rotation gear set.

Figure 2 shows the configuration used to control the angular direction of each thruster, which is set by the servomotors (in red) coupled to the support axes by gearboxes (yellow). Therefore, each motor can assume an individual rotation angle composing the forces $Fx_{1},Fx_{2},Fx_{3},$ and $Fx_{4}$.

## 3. Initial Hull Definition

In order to define the shape of the hulls best suited for the ASV design, different shapes were evaluated qualitatively in a CFD analysis. This study was performed using SOLIDWORKS Flow Simulation, which allows easy integration with the 3D CAD platform used for designing the hulls, as well as relatively quick simulations. There are limitations to the software capabilities, such as the restricted options for the turbulence models and the impossibility to move the hull dynamically during the simulations, to compensate for buoyancy changes and the hydrodynamic pitch moment. These limitations impact the final hydrodynamic forces and wave formation, but still allow a qualitative comparison of the hull shapes considered.

**Figure 3:**
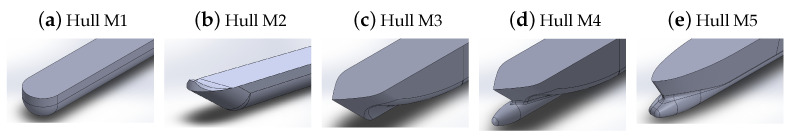
Study of hull shapes.

In Figure 3, it is possible to see the five geometries analyzed. All of the proposed hulls were designed to have a displaced water volume that could counterbalance the expected vessel’s weight with a safe margin. Hull M1consists of a very simple geometry, in a cylindrical shape with the ends in 1/4 of the sphere shape. Hull M2 has a similar design, except for its angular bow, which aims at smoothing the flow displacement, reducing the drag, and providing a positive hydrodynamic pitch to compensate for the motor’s pitch on the vessel’s topdeck.

In order to have a better arrangement to place the batteries and electronic components, Hull M3 with a more rectangular cross-section was proposed. Moreover, to reduce the frontal drag of this new cross-section, a bow with a keel was proposed. Hull M4 was an upgrade of Hull M3 with a bulbous bow to reduce the frontal drag even more. A bulbous bow creates small waves as the vessel moves, which can interfere destructively with the waves created by the remainder of the vessel, reducing resistance.

In this same direction, a more complex structure as shown in Hull M5 was proposed. In this case, it was observed that Hull M5 had a thinner cross-section than the previous hulls, and the addition of the bow bulb was more integrated with the hull.

### 3.1. Simulation Configuration

CFD simulations can be quite expensive, and much of the computational effort is related to the number of cells contained on the mesh. Even when considering a qualitative analysis, a balance is necessary between the desired precision of the results and the time spent on the simulations, minimizing the occurrence of convergence errors that may jeopardize the quality of the analysis.

**Figure 4:**
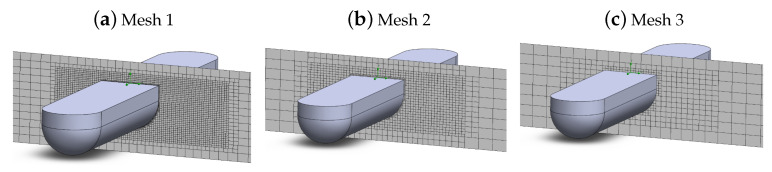
Mesh details.

In this context, three different meshes were generated: one with a high degree of refinement (Mesh 1), one with an intermediate cell size (Mesh 2), and one with a coarser refinement (Mesh 3). In Figure 4, it is possible to observe the general appearance of each mesh, as well as the refined region near the hull, to better capture the quickly changing parameters of flow pressure and velocity near the surface.

In this direction, simulations were carried out to generate comparative parameters between each one. The results obtained are shown in Table 1, where it is possible to notice the great discrepancy in the computational time for each of the cases analyzed, as shown in Table 1.

**Figure 5:**
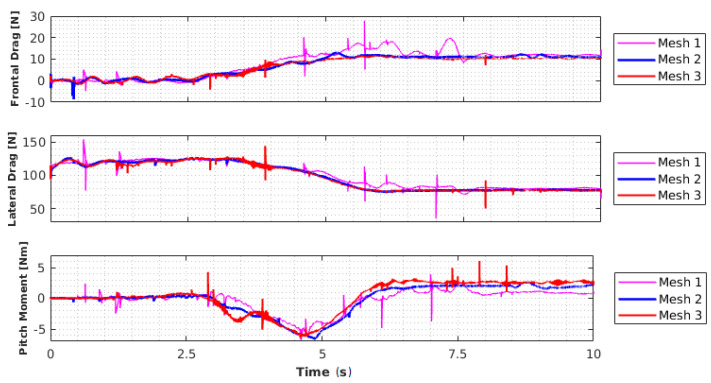
Analysis of forces and moments.

Figure 5 shows the results of the forces and the moment of pitching obtained with the analyses for the different meshes.

It is possible to notice that all the results in Figure 5 converge to a final value at the end of the 10 s of simulation. The results did not show too much divergence from each other; however, it is clear that for the coarser mesh, the peaks (which represent convergence errors) are more frequent. The only parameter that obtained significant differences in its final values was the pitching moment, which, depending on how coarse the mesh was, can present a magnitude of up to 71% lower than that of the more refined mesh. Mesh 2 represents a good balance between computational cost and the consistency of the obtained results, and its structure was replicated in the other analyses elaborated in this section.

### 3.2. Simulation Results and Hull Definition

Once the mesh was defined, the hull shapes were simulated under two possible operating conditions of the vessel: the first with speed in the longitudinal direction of the vessel equal to 2 m/s and the second with the same longitudinal speed, but with a lateral speed of 0.5 m/s. The simulation results are presented in Table 2, where a comparative analysis of the forces and moments is presented.

Considering that the propulsion control can rotate all the way in the horizontal plane, a low resistance to sway is shown to be a great asset in the vessel’s maneuverability. The right balance between frontal drag and lateral drag must be achieved. Other parameters, as described in Table 2, shall be analyzed as well, such as the pitch moment and the yaw moment. A more neutral yaw moment reduces the effort of the control and propulsion to perform a strict trajectory.

When all these things are put in balance, the advantage of Hull M2 is apparent, even when compared to Hull M5, which had the lowest frontal drag. Furthermore, a smoother surface as Hull M2 is a great advantage in shallow water operation and avoids algae, branches, river bottom rocks, or other debris getting stuck to the hull, allowing the vessel to move more freely. Other key features of this hull that make it a good choice for the design are the simplicity of its shape, which can be more easily manufactured when compared to others, and the angular bow, which represents a passive method of dynamically balancing the pitch moment of the aerial thrusters using hydrodynamic pressure.

## 4. Study of Sensor Positioning

This section presents the studies to justify the definition of the ADCP position so that the presence of hulls influences as little as possible the relative flow speed. As a consequence, the sensor can measure the water velocities more precisely.

The studies were also carried out with CFD, which is a powerful tool for modeling complex physical phenomena, especially when used alongside practical tests, validating the computational results [25]. The method allows simulating conditions similar to those encountered in typical ASV missions, and the processed flow data may then be used for determining the optimal sensor position.

In this regard, after presenting the necessary settings for the CFD simulation, two types of analysis were done: the first one refers to the wave elevation around the hulls and their wake, and the second one is dedicated to estimating the flow speed variation around the vessel, especially in areas close to the water surface, where the ADCP might be located.

### 4.1. Problem Definition

**Figure 6:**
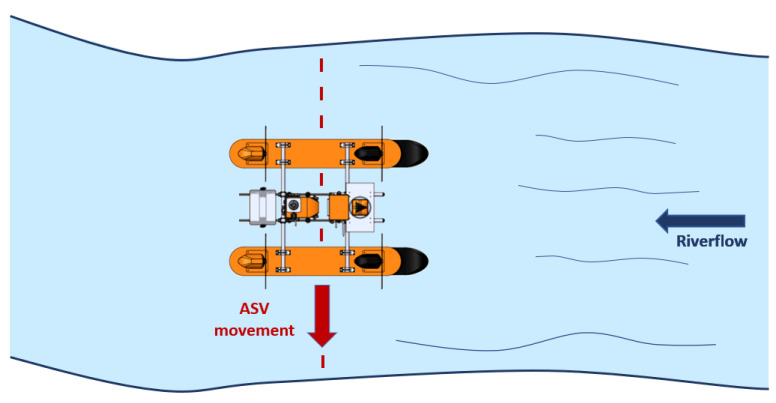
Crossing direction considering the river flow.

The design of the proposed ASV took into consideration multipurpose missions; however, the main goal was to optimize the flow curve (FC) of rivers. The problem with this activity is that, most of the time, extreme events in Brazilian rivers, such as large floods during the rain season change the FC profile, and as consequence, all related mathematical models start to provide imprecise forecasts. During these floods, although the water flow in the extended margins is shallow and slow, it is severely increased in the river’s main course [26,27]. Therefore, it is necessary to overpass a shallow flooded area before starting the ADCP measurements. In such a scenario, wave reflections in shallow waters are not considered in the simulations as it is not a valid operational issue. Moreover, as the proposed ASV has three DOFs, it is designed to cross the river longitudinally as shown in Figure 6; the water inflow go directly to the sensor, and the wave pattern generated by the hulls has no effect on the measurements.

Therefore, the topology is designed to cross shallow flooded waters in order to successfully take precise measurements in the main river’s course while keeping the sensor enclosed by the hulls for protection against floating obstacles. Finally, as a shallow water boat, several other inspection missions could be accomplished, such as water and wild life monitoring [28].

### 4.2. CFD Configuration

In this study, the software chosen for fluid calculations was OpenFOAM, an open-source code with the C++ programming language, based on the finite volume method, which has been widely used in the literature [29,30,31].

To carry out the studies, it basically needs four steps. The first one consists of inserting a 3D CAD simplified boat model into a two-phase computational domain containing water and air, while also specifying mesh motions and boundary conditions. The mesh motions are related to the allowed DoFs in the calculations. In these analyses, only heave and pitch motions are permitted, as they are essential for the vessel to adjust its water level dynamically (compensating its weight, its buoyancy, and the dynamic pressure of the flow), which in return affects the running water dynamics.

The boundary conditions are responsible for creating the relative movement between the hull and the flow. Water and air are set to stream with constant speed from the inlet to the outlet of the computational domain, flowing in the opposite direction to the one the hull is pointing.

**Figure 7:**
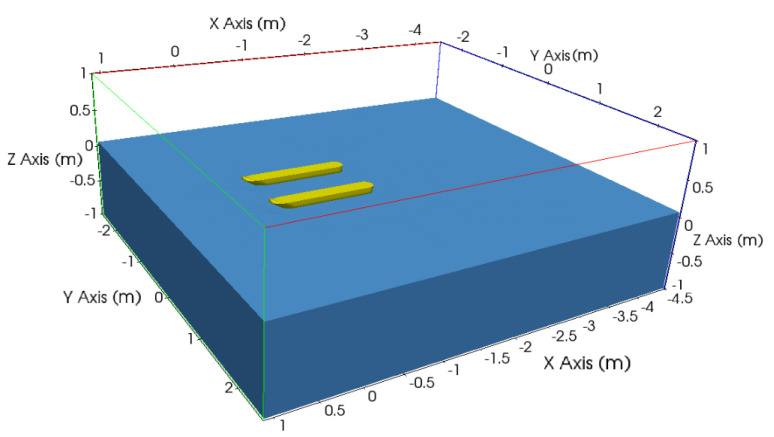
Complete computational domain.

From analyses in which there is flow symmetry with respect to a plane, a single catamaran hull can be simulated and consequently save the computational cost. Figure 7 shows the illustration of this first stage, with a complete computational domain (consisting of the part where the calculations were made and its symmetric part, used only for visualization).

**Figure 8:**
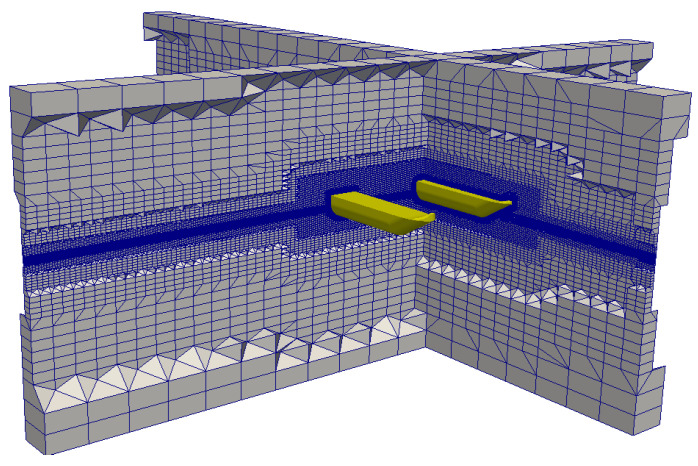
Representation of the mesh used in the complete computational domain.

**Figure 9:**
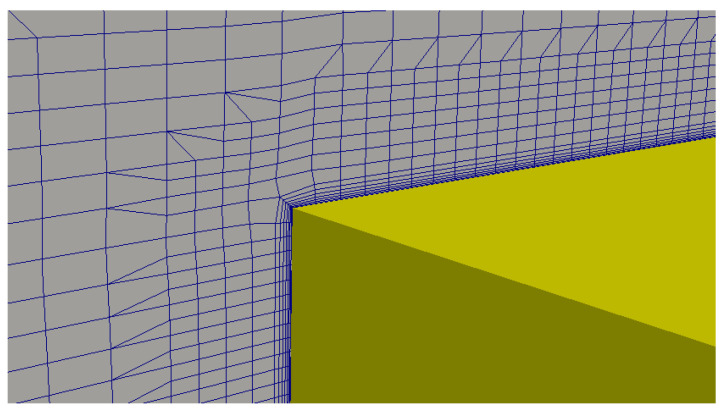
Mesh details around the hull.

In the second step, the domain is discretized into small cells (see Figure 8), where the partial differential equations are solved. A mesh convergence study was performed in each computational domain simulated (in the same fashion as the one shown in Section 3.1), and a final mesh with refinement zones near the water plane and the hull showed a nice trade-off between computational cost and physical fidelity. This refined area was important to assure accuracy when calculating wave propagation and flow velocities near those areas. In the hull vicinity (where pressure and velocity gradients are high), the use of thin prismatic cell layers helped obtain more reliable viscous damping parameters. Figure 9 shows an example of the use of thin prismatic cells around the hull. The first layer is set to have a dimensionless wall distance of 15 and seven consecutive sheets with an expansion ratio of 1.3. Symmetry along the X plane (on the Y-axis origin) halves the number of cells analyzed in the computations, which stay in the order of 6,040,000.

The third step consists of the actual computations of the differential equations that govern the fluid movement, which was done with the aid of the interFoam solver. The Unsteady Reynolds-Averaged Navier–Stokes (URANS) method was used to compute the pressure, velocity, and turbulence fields in each cell of the domain. Using unsteady RANS can help the solver get the correct wave propagation without the need for special numerical treatments [32]. The chosen method requires turbulence parameters to compute the Navier–Stokes equations, and the k−ω turbulence model has been one of the most used methods in ship hydrodynamics papers recently [33], due to its robustness and simplicity. OpenFOAM allows a modified version of this model, the k−ωSST−SAS, to be used alongside the URANS method, and for this reason, it was chosen as the turbulence closure in the calculations. The interFoam solver uses the URANS method along with the PIMPLEalgorithm (which couples equations of momentum and mass conservation in an iterative procedure) to compute the transient flow of the analyzed catamaran.

Euler time integration was used for the domain, and a Crank–Nicholson second-order time integration was used for the hull movements in pitch and heave. The time steps were calculated dynamically by the solver, always ensuring that the Courant number would be below 0.9. This guarantees that the information in the flow passes correctly from each cell to its neighbors, taking the mesh size and flow velocities into consideration throughout the entire domain. The typical total processing time until the transient flow’s convergence was about nine days (778,000 s) running on a computer cluster with 16 cores.

After the convergence of the results, the fourth step begins the post-processing. It consists of the computed information handling, in order to extract the best of the data results for each study case. The Paraview software uses processed flow field information to enable visualization of the cell variables (e.g., pressure, velocity, turbulence parameters) throughout the mesh with the aid of streamlines, flow variable thresholds, and mesh slices. Flow characteristics such as the wave profile can then be analyzed and, when compared to practical tests, used to verify the ideal sensor location.

### 4.3. Results

The results of the post-processing phase are presented in this section, both in quantitative data and through the use of flow visualization tools.

#### 4.3.1. Hydrodynamic Model Parameters

Following the CFD configuration methodology described in Section 4.2, different simulated conditions were analyzed. The results of the hydrodynamic forces and moments on the hull were verified for several flow velocities in the surge, sway, and yaw rotation, each of them being analyzed separately. A quadratic response curve correlating the input velocity to these forces was generated for each DoF, providing the parameters of the hydrodynamic damping matrix ($N(\nu_{r})$) of the model (as seen in Section 2.1). In a similar fashion, a sinusoidal movement was imposed on the hull along both its X- and Y-axis. The hydrodynamic forces acting on the vessel in these analyses were correlated to the acceleration of the body, providing the added mass parameters of the inertia matrix (M) of the model.

The model parameters obtained in this section are summarized in Table 3. These parameters can be used for implementing a dynamic control of the ASV, and therefore are needed for autonomous missions of the vessel.

#### 4.3.2. Sensor Positioning

**Figure 10:**
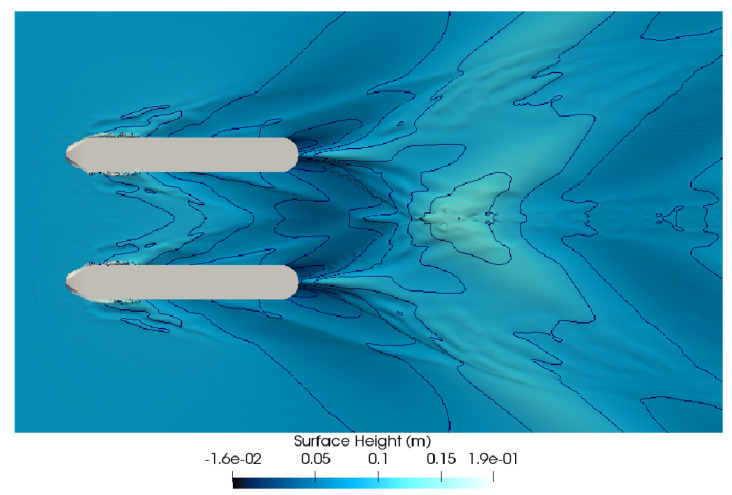
Simulated wave pattern at 2 m/s (Fr=0.54).

The analyses for sensor positioning were based on the velocity of 2 m/s (consisting of a Froude number of 0.54), a vessel speed that was considered representative of its typical mission. A top view of a simulation in this condition is shown in Figure 10, focusing on the height of the water-air interface and the wave formation as calculated by the software.

It is possible to observe that after the rear half of the ASV, the flow between the two hulls gets considerably disturbed, with a wave through lowering the waterline position until the flow from both sides converges on a large wave crest. The front half has a tendency to maintain the waterline height approximately the same as the undisturbed flow.

**Figure 11:**
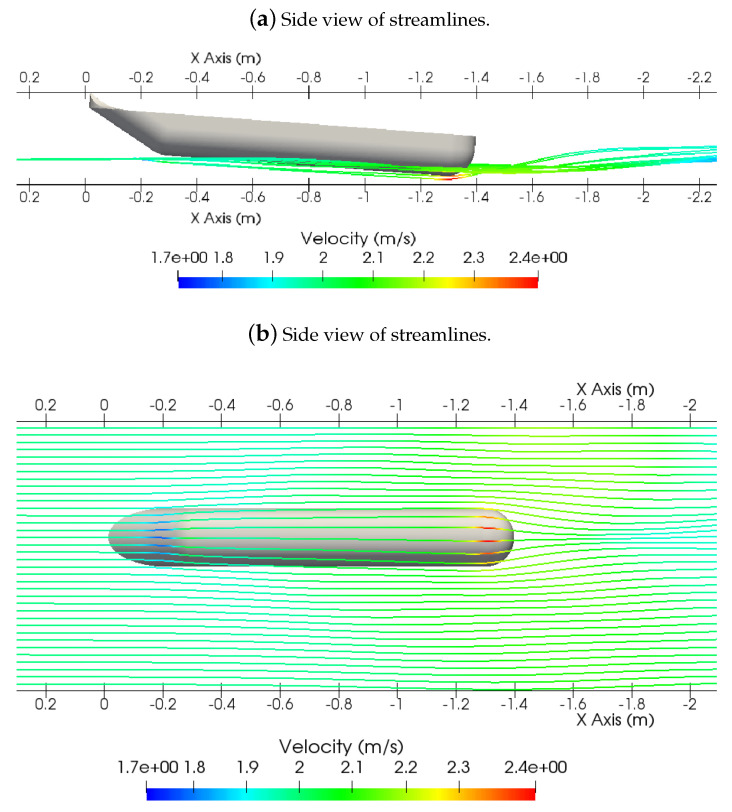
Streamlines displaying information of the flow velocity in an analysis with a Froude number of 0.54.

As the boat speeds up, the bow tends to increase its height as it tilts its angle, due to the water impact on its angular bow. Figure 11a shows the boat tilt and the flow streamlines around the hull. Once again, it is possible to notice that the vertical position of the flowing particles remains relatively unaltered until they get to the middle of the ASV. Figure 11b shows the streamlines with colors marking the velocity viewed from the bottom of one of the hulls. The streamlines show the pathway that water particles are likely to go through, and it is possible to see that they remain relatively close to the vessel’s body flowing in an orderly manner, as is expected of a non-turbulent stream.

**Figure 12:**
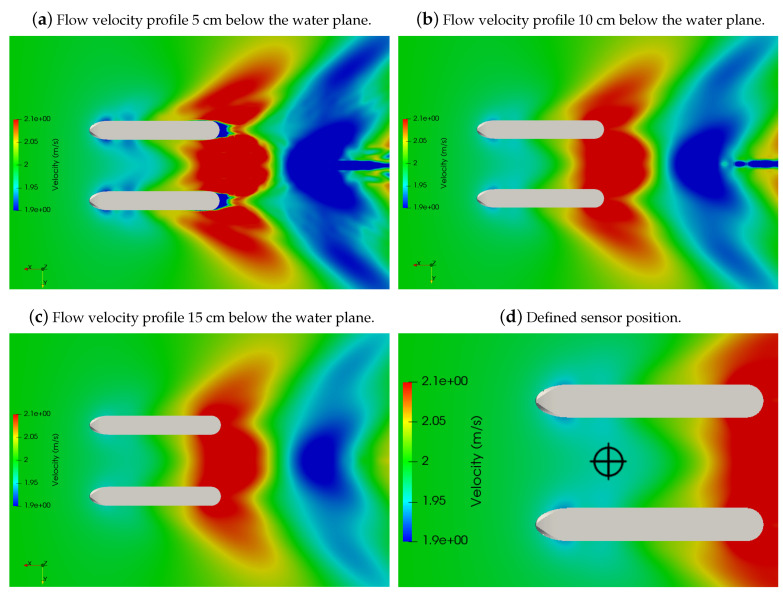
Flow velocity profile in different planes below the water line and the defined sensor position.

Figure 12a–c shows the diagram of the velocity in a plane 5, 10, and 15 cm below the water line, respectively. The interference caused by the hull on the flow is more significant in the section corresponding to the rear half of the vessel, where the water speed can have variations greater than 5% of the velocity on the undisturbed stream.

### 4.4. Discussion

The simulations presented aimed to find a perfect spot to place the ADCP sensor. It must be well insulated from debris that can be carried with the flow and avoid it bumping the floor or a rock in the riverbed. It shall be placed in a region where the velocity is not considerably disturbed by the presence of the hulls and the elevation of the waves does not cause the interference of the sensor measurement. The sensor position was defined based on a trade-off among these requirements.

The place in Figure 12d presents a particular place where the velocity variation is below 2%. Furthermore, the “v” shaped waves formed in the bow hulls encounter each other right after the sensor, causing a slight elevation, shown in Figure 10. These wave formations will avoid a valley downstream from the sensor, thereafter reducing the sensor drag. The chosen point is very well insulated from outside obstacles once it is placed in the middle, between the hulls. Furthermore, if placed in a position further upstream, the adcp sensor may modify the vessel’s center of gravity beyond its design point, with consequences on the drag, pitch moment, and yaw dynamics.

It was also observed that for the higher speeds analyzed, the primary wave crest moves backward, towards the stern, which can increase the flow interference in this area. Considering that greater magnitudes of water velocity lead to a greater relative percentage of expected error in disturbances caused by wave turbulence, this interference could be responsible for more significant changes in the sensor readings, if it was placed in this position.

## 5. Final Design and Data Validation

**Figure 13:**
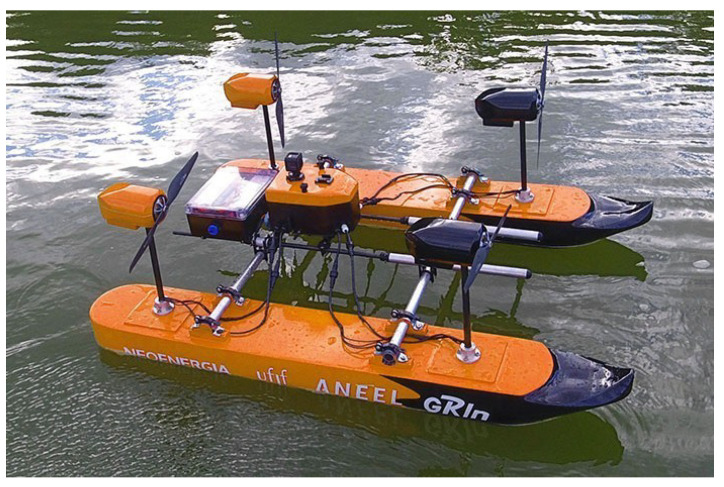
Real view of the developed catamaran.

Based on the requirements seen in Section 2 and the aforementioned CFD studies, the USV AERO4RIVER was developed and manufactured, as shown in Figure 13. It is possible to observe the shape of the hull (M2) defined in Section 3, the azimuth aerial propellers, as well as the aluminum support for the adaptation of the ADCP, according to Section 4. Note that the vessel obtained allows navigation in shallow water and navigation in the three fully controllable DoFs, from the composition of the propeller forces.

Table 4 shows the ASV’s physical characteristics and dimensions. The final specifications of the ASV show that the remaining requirements set for the design (maximum speed, weight, size) were all met. It can be seen from Table 4 that the vehicle achieves a maximum speed of 3.05 m/s with a 25 kg weight. The vehicle is compact enough so that transporting it to a mission is not much of a burden.

**Figure 14:**
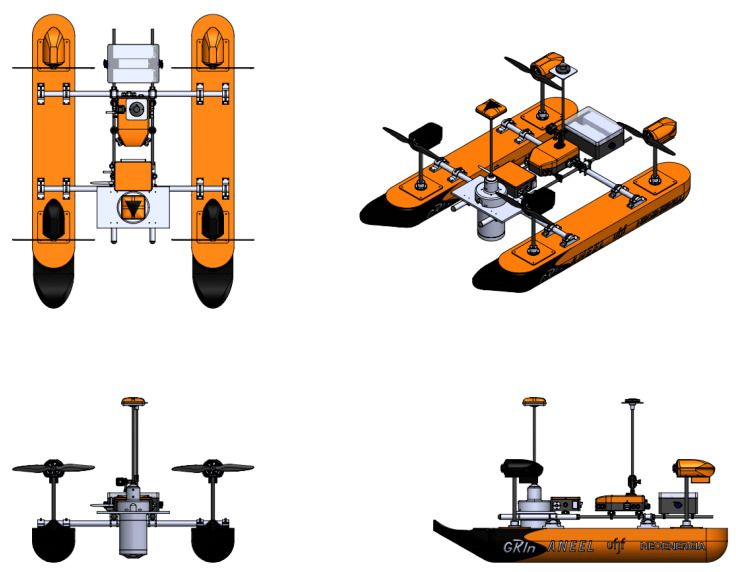
ASV technical drawing.

Figure 14 presents the designed CAD model of the vessel with the ADCP sensor installed in the position defined in Section 4.4. The ADCP is attached to the boat in a way that its position can be easily lowered or raised, according to the conditions of the environment being measured (presence of eddies in the currents, for example). In ideal conditions, a position between 5 and 10 cm below the surface level ensures that the sensor always stays underwater and has a velocity variation caused by the hull water displacement as low as 2%, as discussed in Section 4.4.

### 5.1. Overall Control Structure

This ASV is a very stable and controllable dynamic system for the water system taken into account, so it is not the purpose of this work to study or compare more sophisticated control techniques.

**Figure 15:**
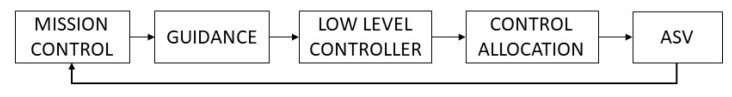
Overall control structure.

The navigation structure shows in Figure 15 is divided into subsystems separating the vehicle guidance and control problems into outer and inner loops. The positioning loop controls the vehicle’s inertial displacement; the lower level is responsible for controlling the yaw and speeds. Finally, the control allocation is responsible for transforming the virtual control forces into real actions. A deeper description of the control levels and control allocation methodology can be found in [34].

### 5.2. ADCP Communication System

**Figure 16:**
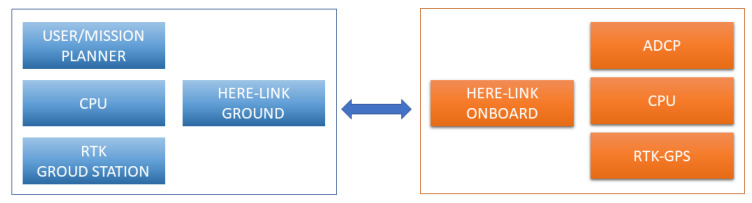
Communication block diagram.

The ADCP, as well as the vessel use a ground communication system in addition to an internal storage system. This system is also responsible for sending corrections for the GPS RTK. In this way, the block diagram representing the data communication of the system can be seen in Figure 16.

The RTK GPS base makes corrections available to the ground control station, which in turn sends them to the ADCP. In addition to receiving the corrections, the ADCP sends back a signal with basic data to the ADCP monitor and stores the best quality data in its internal memory.

### 5.3. Comparisons between CFD and Practical Tests

In order to validate the USV design and the CFD analyses, tests were carried out with the vessel, and the wave patterns it formed while moving in the water were captured from aerial photos taken from a Phantom drone. The tests were performed with the vessel operating at 2 m/s, the same speed that was set in the CFD simulations, as it is considered to be the velocity of a mission.

**Figure 17:**
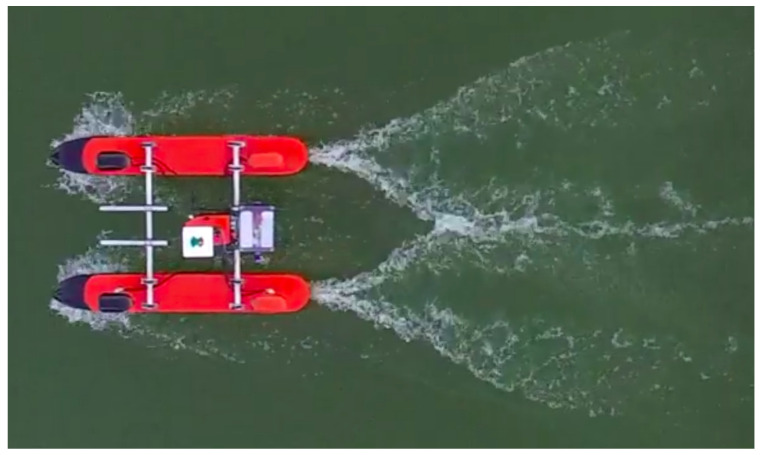
Wave pattern at 2 m/s (Fr=0.54) in the practical test.

In Figure 17, it is possible to see one of these aerial photos, where the wave pattern is clearly noticeable. In this figure, we observe the presence of the hull in the flow presenting a wave pattern with a “v” shape, as shown in Figure 17. This pattern is particularly similar to the wave pattern shown in the simulated analysis of Figure 10. Furthermore, wave peaks on each side of the hull bow can be seen, and the formation converges for the central position of the boat between the hulls, where, after those, the ripples of valleys and crests are formed. In the stern part, the lowest elevations are formed, showing a huge “v”-shaped wake with the highest elevations.

**Figure 18:**
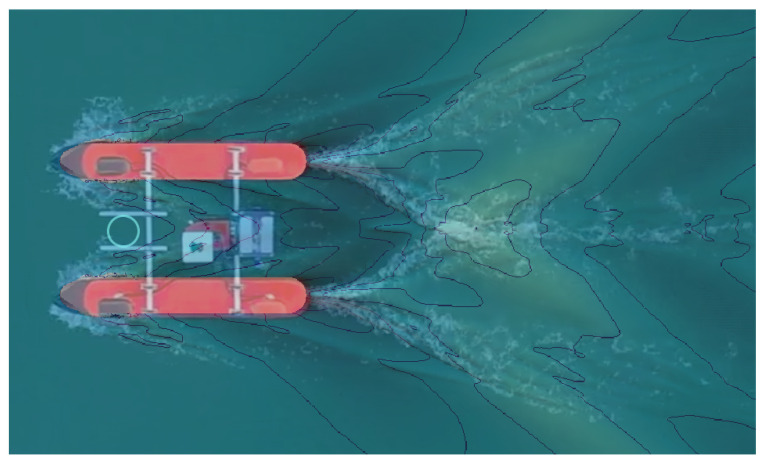
Wave pattern at 2 m/s (Fr=0.54) in the practical test image overlayed with the simulation image.

In Figure 18, an image overlapping the simulation and the experimental results is shown, making clearer their similarities. The ADCP sensor is designed to be attached to the frontal support (as seen in Figure 12d and Figure 14), in a region practically unaffected by the hull disturbances on the flow. This region is marked in Figure 18 by the blue circle in the front part of the vehicle.

As can be seen, the results match almost perfectly. Aside from the small flow irregularities that the mesh size was not able to capture, all of the characteristic features in the wave pattern were well replicated in the CFD results. The similarities between the simulated and real conditions are important for multiple reasons. They can contribute to validating the analysis parameters, as well as the mesh definition. Furthermore, the wave profile is intrinsically dependent on the pressure and velocity fields across the domain. Consequently, if the simulation accurately captures the flow behavior, it is also able to inform about the velocity profile reliably, helping to validate the definition used for placing the ADCP sensor on the vehicle.

### 5.4. Comparisons between the Model Estimated and the Practical Tests

To evaluate the mathematical model estimated by the CFD, three new tests were developed to compare the vessel’s behavior under normal operating conditions. In the first scenario, the vessel is exposed to frontal movements with a wide variation in operating speeds, while in the second scenario, small yaw movements are also added. In the last scenario, the traditional zigzag movement is introduced to the vessel, imposing rapid yaw dynamics and lateral movement.

**Figure 19:**
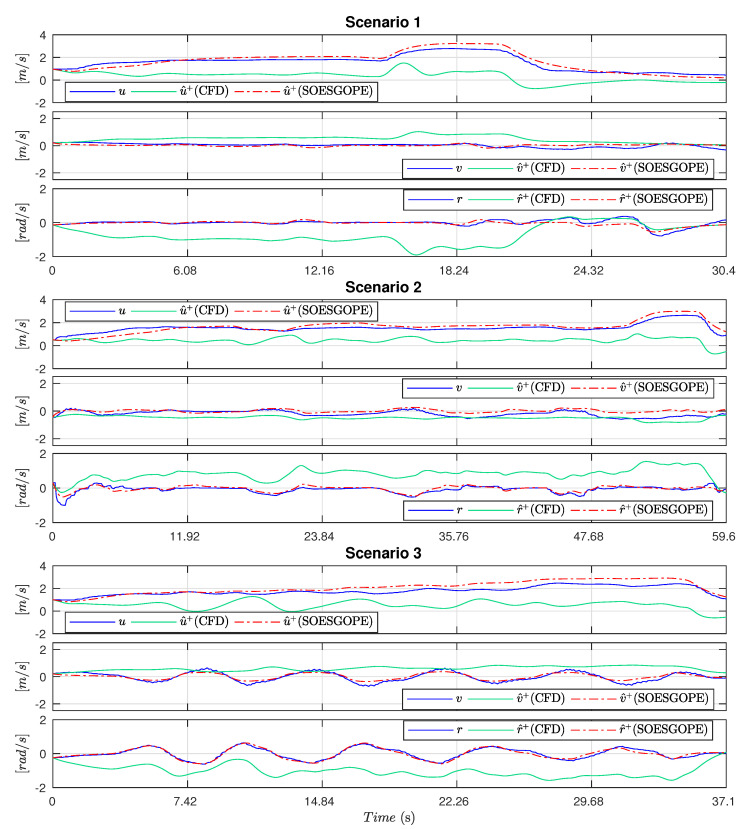
Comparisons between the model estimated and the practical tests.

In this case study, the parameters initially estimated were improved by the optimal parametric estimation method called SOESGOPE [35,36], with the intention of adding operating characteristics not yet covered by the initial CFD model. The results obtained are presented in Figure 19, while Table 5 shows the Root Mean Squared Error (RMSE). Furthermore, Table 6 presents the CFD parameters and improved ones.

Analyzing the results in Figure 19 and Table 5, it is possible to note the quality of the model obtained by SOESGOPE, which by improving the initial estimate, was able to satisfactorily represent the dynamics of the vessel in the proposed scenarios. It is also possible to apprehend the validity of the initial estimate of the CFD, which, despite the divergences presented, managed to simulate the behavior of the main dynamics and provide important information for a more accurate estimation.

## 6. Conclusions

A methodology combining hull design and sensor placement was presented in this work. The correlation with experimental results was also shown and discussed.

The CFD method to determine the best position for placing the sensor is shown to be a great asset in the design and instrumentation process. Other types and usages of sensors could benefit from a similar procedure.

The biphasic simulation showed a good correlation with the experimental results, such as the wave elevation and its fluids interface pattern. The post-processing tools of the CFD resulting in graphics showing the velocity at different depths were truly relevant for determining the best ADCP position. Furthermore, the results revealed that complex simulations can be performed at a relatively low computational cost. Furthermore, the procedure can be used while the vessel is still in the design phase, without a built prototype to be tested. This could help with incorporating the sensor positioning in a preliminary design phase of the vessel’s design, therefore optimizing the layout of the vehicle.

## Figures and Tables

**Table 1:** Mesh analysis data.

	Mesh 1	Mesh 2	Mesh 3
Number of Cells	997,035	199,030	34,730
Analysis time	57:43:03	3:42:45	0:17:05

**Table 2:** Mesh analysis data.

		M1	M2	M3	M4	M5
	Hull height (mm)	150	150	150	200	200
	Hull draft (mm)	66	85	92	79	86
Frontal velocity: 2 m/s	Frontal drag (N)	11.65	7.61	8.01	7.41	6.37
	Buoyancy (N)	95.43	60.71	79.22	83.15	100.79
	Pitch moment (N.m)	0.00	5.75	4.18	2.57	0
	Wave height (mm)	151	95	94	94	117
Frontal velocity: 2 m/s Lateral velocity: 0.5 m/s	Frontal drag (N)	13.02	7.60	8.27	9.01	7.56
	Lateral drag (N)	11.61	9.80	24.67	23.52	21.52
	Buoyancy (N)	67.44	47.70	67.84	64.01	77.35
	Pitch moment (N.m)	2.36	6.29	4.61	4.00	0.73
	Yaw moment (N.m)	−5.25	−3.81	−5.33	−5.20	−10.30
	Wave height (mm)	144	115	155	166	138

**Table 3:** Parameters estimated by CFD and SOESGOPE.

Parameter	Value	Unit
$|X_{\dot{u}}|$	2.00	kg
$|X_{u}|$	0.40	kg/s
$|X_{|u|u}|$	7.30	kg/m
$|Y_{\dot{v}}|$	26.00	kg
$|Y_{v}|$	1.88	kg/s
|$Y_{|v|v}|$	31.20	kg/m
$|N_{r}|$	0.90	kg × m${}^{2}$/(s × rad)
$|N_{|r|r}|$	6.60	kg × m${}^{2}$/rad${}^{2}$

**Table 4:** Vehicle description.

Propellers	
Building material	Carbon fiber
Diameter	25 cm
Pitch	0.5”
**Engines**	
Mass	0.258 kg
Maximum electrical current	20 A
Number of units	4
Rotation/Volt (Kv)	1000
Technology	Brushless
**Individual Thrust Set**	
Maximum thrust	21 N
Thrust coefficient (K1)	0.021
**Battery**	
Cells number	6
Individual weight	1.845 kg
Nominal voltage	22.2 V
Technology	Lithium
Total charge	16,000 mAh
**Assembled Vehicle**	
Center of gravity to propeller distance	0.586 m
Hull width	0.2 m
Hull length	1.4 m
Hull height	0.162 m
Hull distances	0.55 m
Medium operating weight	25 kg
Maximum linear speed—surge	3.05 m/s
Maximum angular speed—yaw	2.32 rad/s
Number of batteries	2

**Table 5:** RMSE results.

	Scenario 1	Scenario 2	Scenario 3
CFD	$2.4741\times 10^{6}$	$8.7231\times 10^{6}$	$4.6306\times 10^{6}$
SOESGOPE	$0.0029\times 10^{6}$	$0.1754\times 10^{6}$	$0.0033\times 10^{6}$

**Table 6:** Hydrodynamic parameters.

Parameter	CFD	SOESGOPE	Unit
$|X_{\dot{u}}|$	2.00	14.15	kg
$|X_{u}|$	0.40	3.75	kg/s
$|X_{|u|u}|$	7.30	5.24	kg/m
$|Y_{\dot{v}}|$	26.00	8.24	kg
$|Y_{v}|$	1.88	17.63	kg/s
$|Y_{|v|v}|$	31.20	292.62	kg/m
$|N_{\dot{r}}|$	−	8.22	kg × m${}^{2}$/r(rad)
$|N_{r}|$	0.90	3.63	kg × m${}^{2}$/(s × rad)
$|N_{|r|r}|$	6.60	5.92	kg × m${}^{2}$/rad${}^{2}$

## Data Availability

Not applicable.
